# Hardening and Tempering Instruments

**Published:** 1882-02

**Authors:** Chas. A. Blake

**Affiliations:** Philadelphia.


### ARTICLE VI.

### *Hardening and Tempering Instruments.*

BY CHAS. A. BLAKE, OF PHILADELPHIA.

We frequently hear members of the profession, particularly those remote from the manufacturers, say, " I can get along with everything but *tempering*." Really it is the most simple of all operations they have to perform outside of the operating room; although there are some who would mislead them by trying to invest it with unnecessary difficulties or secrecy.

The object of the present article is to give some plain and simple directions for hardening and tempering. To begin, then, we know there are two kinds of steel, good and bad; of the latter little need be said, except—discard t. Some will say, however, how are we to distinguish the good from the bad? This is easily done by breaking a piece of steel; if the fracture presents a course or glitterng appearance, do not use it. If the steel is good, the racture presents a fine, close grain, of a grayish-white color, the whiter the better, if the grain is fine.

The finer the steel, the lower the heat required to harden it; fine steel being more readily burnt than the commoner qualities. It is improved by being well hammered at a low red heat, each time before hardening.

If the instrument to be treated is an excavator, it must first be annealed, or softened; this may be done in the flame of a spirit lamp, or what is better, a bunsen burner where gas is available. Hold the part to be softened in the flame till a dull, red heat appears on it, then let it cool slowly. When ready, file the point to the required size, and bend to suit; then heat it in the flame till it assumes a bright red, but not sufficient to cause it to *sweat* or blister, taking care that the point does not get too hot before the shank of the instrument is brought to the proper heat. The red heat should extend about an inch from the point. When sufficiently hot, plunge it into cold water; if the water is slightly salt, it is somewhat improved, but this is not absolutely necessary.

Next, carefully rub the part hardened, on a piece of emery paper, or anything that will make it white. Having brightened it, hold the thickest part of the shank in the flame till it becomes blue, and gradually draw it towards the point, which should be held against some cold substance (the end of a file, or even a common nail will answer the purpose) to prevent it getting blue. Allow the shank to become blue all along to the bend; the blade will now be straw color, at which moment dip it in water. The blue color of the shank leaves it hard enough to resist any lateral force, while the blade being straw color, it will be sufficiently hard to cut. It is now ready for polishing.

In the case of a plugger—having softened it, file and bend to the required form, serrat it according to its requirements, with a very sharp-edged file; heat to the same degree of redness as for excavators, plunge into cold water, rub bright as before, then hold the shank in the flame until blue, gradually drawing it towards the point, allowing the latter part to become a reddish-purple, then cool and polish.

For nerve instruments which require a spring temper, it is necessary to heat to a bright red, and immerse in oil; when cool, draw it through the flame till the oil ignites and burns out, when it will be found elastic.

Chisels for cutting enamel should be heated to a dull red, and well hammered; having done this, file to the required form, making the level, or cutting-edge, *short*; an edge of this description may be left very hard without danger of breaking. Heat as directed for excavators, harden in water, temper the shank blue, and let the straw color of the blade get lighter, almost white near the edge. Chisels treated in this manner may be quite thin, and will be found preferable for most operations, being strong enough to withstand any ordinary pressure.

It must be borne in mind that both iron and steel will take all the colors referred to above, by heat, whether hard or soft; so that it is best to be sure of *hardness* before the tempering. This is easily done by passing a file over the part that has been heated and cooled. No instrument will stand if left perfectly hard.

The best form of burner for these operations is the Bunsen, as it gives a *clean* flame, depositing no soot, so that the colors are readily seen. A blowpipe may be used, but great care must be taken to avoid overheating the points. Steel brought to a *white* heat is destroyed.—*Items of Interest.*

